# Effect of Zinc Excess on *Sinapis alba* L. Seed Yield, Biochemical Parameters, and Potential for Further Processing

**DOI:** 10.3390/plants15121778

**Published:** 2026-06-09

**Authors:** Natalia Repkina, Svetlana A. Murzina, Viktor P. Voronin, Yulia Batova, Elena Ikkonen, Ekaterina Antonova

**Affiliations:** 1 Institute of Biology of the Karelian Research Centre of the Russian Academy of Sciences (IB KarRC RAS), 11 Pushkinskaya St., 185910 Petrozavodsk, Russia; murzina.svetlana@gmail.com (S.A.M.); voronen-viktor@mail.ru (V.P.V.); batova.krc@mail.ru (Y.B.); likkonen@gmail.com (E.I.); antonova88ep@mail.ru (E.A.); 2 Institute of Physical Education, Sports, and Tourism, Department of Life Safety and Health-Saving Technologies, Petrozavodsk State University (PetrSU), 33 Lenin St., 185910 Petrozavodsk, Russia

**Keywords:** seeds, lipids, zinc, biodiesel properties, recycling

## Abstract

Excess zinc (Zn) has an important effect on seed yield and quality, as well as the possibility of subsequent processing. In this study, we investigated the effect of excess Zn concentrations (50, 100, and 150 mg kg^−1^) in the substrate on the seed yield and selected biochemical parameters of white mustard (*Sinapis alba* L.) seeds. The following parameters were investigated: seed yield, individual lipid classes were analyzed using high-performance thin-layer chromatography (HPTLC); gas–liquid chromatography with mass-selective detection (GS-MS) was used to analyze the fatty acid (FA) profile; and trace elements were detected using inductively coupled plasma-mass spectrometry (ICP-MS). It was found that Zn at concentrations of 100 and 150 mg kg^−1^ caused a decrease in the number of *S. alba* pods, and the 1000-seed weight also decreased at a Zn concentration of 150 mg kg^−1^. The Zn concentration in seeds from plants grown on contaminated substrate was higher than the control values and government standard thresholds. Zn at concentrations of 100 and 150 mg kg^−1^ caused a slight increase in the content of triacylglycerols (up to 1.35–1.36% dry weight) and the total content of unsaturated FAs along with a decrease in saturated and total FAs. Zn at a concentration of 50 mg kg^−1^ stimulated an increase in the total FA content. A high erucic acid content in *S. alba* seeds was observed in all studied variants. A mathematical model was used to evaluate the physicochemical properties of biofuels. The parameters relating to the FA composition of seeds of plants grown on a Zn-contaminated substrate did not deteriorate, and in the case of the kinematic viscosity coefficient, they were even improved compared to the control plants and complied with American (ASTM D6751) and European (EN 14214) standards for biofuels. The obtained data indicate a negative effect of high Zn concentrations on seed yield. Changes in the FA composition of *S. alba* seeds may reduce their value for the production of edible oils due to the high content of erucic acid, but they could be used to produce technical oils (biofuel).

## 1. Introduction

An imbalance of chemical elements in the substrate, often caused by heavy metal contamination, negatively impacts plant growth, development, and productivity [1]. Mineral fertilizers are currently widely used to enrich agricultural soils with essential elements, especially in areas exposed to high-risk farming conditions [2]. However, this leads to the accumulation of chemical elements in the substrate, which, in high concentrations, can be toxic [3,4]. Zn, for example, is an essential nutrient that is involved in various biochemical processes in plants, animals, and humans. However, its toxicity can have serious consequences for human health. Several reviews have been conducted on the role of Zn in various processes [3,5,6]. On the one hand, the problem of eliminating Zn deficiency in both soil and plants is currently topical. In this respect, soil supplementation with Zn-containing fertilizers or distant hybridization technology are used to improve plants’ ability to assimilate Zn [7,8,9,10,11]. On the other hand, excessive use of fertilizers and anthropogenic activity lead to the accumulation of Zn and other ions in soils and plants. This, in turn, can cause these ions to accumulate in animals when they are consumed though the food chain [12].

To prevent the accumulation of high concentrations of heavy metals, researchers are currently exploring a promising phytoremediation technology that involves using plants to extract and stabilize metal ions. This technology has been shown to be effective in removing heavy metals from contaminated soil [13,14,15,16]. However, there are still some challenges that need to be overcome in order to fully realize the potential of this technology. One of these challenges is finding suitable plant species that can absorb metal ions from contaminated soil. The *Brassicaceae* family has shown promise in this area, as several species in this family have been found to grow well in contaminated soil and have the ability to absorb metal ions. It is known that a number of plant species of this family such as *Thlaspicaerulescens* [17,18], *Capsella bursa-pastoris* [19], *Brassica juncea* [20,21], and *Brassica napus* [22,23] are resistant to soil contamination, absorbing and accumulating metal ions. This family also includes a number of oilseed crops, in particular, white mustard (*S. alba* L.). Mustard plants and seeds have a wide range of practical applications. For example, *S. alba* flour and bran are known to be rich in phenolic compounds [24], and the oil from these plants is used in rainbow trout feed [25] and improves the transdermal penetration of drugs [26]. Mustard seed extract has bioherbicidal properties [27] and can also be used as a feedstock for biofuel production [28]. It has recently been shown that white mustard can be used in phytoremediation [29,30]. With respect to the relevance of *S. alba* seeds, it is important to understand how their quality changes under the impact of heavy metals. Despite studies investigating the resistance of white mustard to heavy metals [29,31], there are almost no data on their impact on the production and quality of seed. *S. alba* seeds are used as raw material for oil production; therefore, it is important to evaluate the qualitative and quantitative composition of fatty acids and lipids, as their different ratios directly affect the physicochemical properties of the resulting oil [32,33]. However, available data in this area are very limited. Only a few studies have been conducted on the effects of heavy metal ions on the lipid and FA composition in plants [34,35,36] and seeds [37].

Based on the above, this study aimed to evaluate the effect of Zn excess in growth substrate on the yield and quality of *S. alba* seeds, with particular emphasis on their lipid and fatty acid composition, to assess their quality and potential for further processing. We hypothesized that Zn excess in the substrate can affect the composition of lipids and fatty acids in the seeds of white mustard plants, and based on these changes, options for further seed processing are proposed.

## 2. Results

### 2.1. Yield Parameters of S. alba Seeds Under Zn Excess

Along with the increase in Zn concentration in the substrate, the number of *S. alba* pods and seeds and the thousand-seed weight decreased (Table 1). Decreases in the pod number were observed at high Zn concentrations of 100 and 150 mg kg^−1^. However, significant differences in the thousand-seed weight compared to the control were observed only at a Zn concentration of 150 mg kg^−1^.

All Zn concentrations studied had a negative impact on germination energy (Table 2). Seeds formed in the presence of a Zn concentration of 50 mg kg^−1^ demonstrated a decrease in germination rate, but this remained above 90%. When plants were grown at higher Zn concentrations (100 and 150 mg kg^−1^), the seed germination rate did not reach 80%. It should be noted that a germination rate of 90% or more is considered normal for *S. alba* seeds [38].

### 2.2. Trace Element Content in S. alba Seeds Formed Under Zn Excess

The Zn ion content in seeds increased along with the Zn concentration in the substrate (Table 3). This effect is logical. P and Mn ions accumulated in seeds with an increase in the Zn concentration in the substrate (Table 3). A Zn concentration of 150 mg kg^−1^ caused an increase in the Cu content.

### 2.3. Total Lipid Content in S. alba Seeds Under Zn Excess

In the *S. alba* seeds, high contents of waxes, esterified cholesterol, and TAGs were observed in comparison to other lipid classes, regardless of the zinc concentration in the substrate (Table 4). Along with the increased Zn content in the substrate, the TAG/PL ratio also increased, which indicates the predominance of energy metabolism over constructive metabolism. The total phospholipid (PL) content decreased slightly under the influence of Zn at doses of 50 and 100 mg kg^−1^, while the content of DAG, MGDG, and FFA remained virtually unchanged regardless of the Zn content in the substrate (Table 4). An increased wax content compared to the control was observed in seeds from plants grown at Zn levels of 50 and 150 mg kg^−1^.

### 2.4. Fatty Acid Content in S. alba Seeds Under Zn Excess

With an increase in the substrate Zn concentration, a decrease in the content of saturated fatty acids (SFAs) in *S. alba* seeds was observed. The greatest decrease in SFA content (approximately twice) was found in variants of Zn concentrations of 100 and 150 mg kg^−1^ (Table 5). However, the content of unsaturated fatty acids (USFAs) increased in all treatments compared to the control. Consequently, the total fatty acid content increased compared to the control under a Zn concentration of 50 mg kg^−1^, while it slightly decreased under the Zn concentrations of 100 and 150 mg kg^−1^. Several patterns were identified, in particular, an increase in the palmitic acid content and a decrease in the myristic acid content with increasing Zn concentration. It is worth noting a significant decrease in the content of arachidic and behenic acids with increasing zinc concentration. Among the USFAs, an increase in oleic, linolenic, gadoleic, and erucic acids was observed with increasing zinc concentration in the substrate. Also, due to the high content of USFAs, the USFA/SFA ratio was higher in all experimental treatments than in the control.

### 2.5. Proposed Physicochemical Properties of Biofuel

Using the mathematical model described by Pinzi et al. [39] and Sáez-Bastante [40], some parameters based on the FA content were calculated that enabled the physicochemical properties of biofuel obtained from *S. alba* seeds after cultivation on a Zn-contaminated substrate to be predicted (Table 6).

Along with the increase in the substrate Zn concentration, the CN, ν, FP, and CFPP values decreased. Changes in these characteristics can affect the physicochemical properties of biofuel; however, the values comply with the American (ASTM D6751) [41] and European (EN 14214) [42] standards used to evaluate biofuels.

## 3. Discussion

The danger of heavy metal ion uptake and accumulation in plants is not only associated with their negative impact on plant growth and development, but also with reduced seed viability. Poor-quality seed material cannot be used in large-scale farming efforts. Plants implement a wide range of defense mechanisms, including those aimed at limiting heavy metal ion uptake into seeds. However, there is a lack of research on seed quality and viability after cultivation on contaminated substrates.

It has previously been shown that in barley plants grown under conditions of Zn soil contamination, low concentrations of the metal in the soil stimulated seed productivity, but high concentrations negatively affected this parameter [43]. In some species, a decrease in seed viability has been noted. In particular, under pollution conditions, the germination ability of *Atriplex patula* L. seeds decreases; however, the authors observed base protective reactions in plants of the “maternal lineage” under technogenic load. Specifically, there was an increased content of low-molecular-weight antioxidants, activation of DNA repair systems, and an increase in the total content of chlorophylls, with their ratio and presence in the light-harvesting complex maintained [44]. Barley seeds formed by plants grown in substrate with 160 mg kg^−1^ Zn were characterized by lower germination ability, weak initial growth of seedlings, and also lower resistance to high metal concentrations [45]. Our findings are consistent with those of other researchers: specifically, high Zn concentrations in the substrate negatively impact seed production in *S. alba*, as well as seed vigor and germination. It has previously been shown that cytogenetic abnormalities in seeds directly affect their viability [46]. Disruptions to physiological processes in the “mother” plant can also affect the accumulation of essential nutrients in seeds, which could be one of the possible causes of reduced seed viability [45].

It is known that an imbalance of elements in the substrate directly affects the mineral composition of seeds [47]. However, it is still unknown whether assessing the quality of seeds obtained from plants grown on substrates contaminated with heavy metals can facilitate their further processing. Our experiments revealed that a high (150 mg kg^−1^) Zn concentration in the substrate led to increased P, Zn, and Mn content in *S. alba* seeds. The observed increase in the Zn ion content in seeds as its concentration in the substrate increased is logical, but it is worth noting that it was much lower in the seeds than in the plant itself, as we have shown previously [35]. This corresponds with the well-known idea that in the presence of excess heavy metal ions in the substrate, plants implement a range of mechanisms aimed at preventing their entry into the reproductive organs in excessive quantities. It is also known that both synergistic and antagonistic interactions are possible between trace elements [47,48]. For example, synergistic interactions between Zn and P have been shown [49], which was confirmed by our results regarding *S. alba* seeds. It has also been shown that in Arabidopsis plants, in the presence of excess Zn, the amount of Mn and Cu in the roots and aboveground organs decreased [50]. We did not detect a decrease in these elements in the seeds, which may be explained by the fact that changes in the concentration of certain elements in plants in the presence of heavy metals can vary in different plant organs. In general, *S. alba* seeds obtained from plants grown at all studied Zn concentrations met the standards used to assess food safety for oilseeds [38]. Considering the high Zn content in seeds grown in substrate with a Zn concentration of 100–150 mg kg^−1^, the possibility of their use for biofortification in regions with a deficiency of this microelement can be considered.

White mustard is an oilseed crop, and it is important to know its lipid and fatty acid content in order to assess the quality of its seeds and choose further processing techniques. Research has shown that depending on the Zn content of the substrate, the PL content in *S. alba* seeds is approximately 20%, and the TAG content is also high. This is consistent with data reported by Antova et al. [51], obtained from an analysis of the lipid composition of seeds of different *S. alba* varieties. A high TAG content is typical of seeds of many plant species, including oilseeds, as they serve as a primary storage component and energy and carbon reserve. However, there are conflicting data regarding the prevalence of free sterols relative to esterified cholesterol. Some authors note a predominance of free sterols [51], while others report the opposite trend [52]. In our experiments, a high esterified cholesterol content was observed across all treatments. This is likely due to the varietal characteristics of the plants.

When analyzing the fatty acid composition of the seeds, 8 saturated and 11 unsaturated fatty acids were identified. The following acids had the highest content: C16:0, C24:0, C18:1, C18:2, C18:3, and C22:1. A high content of acids such as C18:1, C18:2, C18:3, and C22:1 in *S. alba* seeds has also been observed by other authors, while it is noted that their quantitative content can vary depending on the variety [53] and the seasonal weather conditions [54]. A Zn concentration of 50 mg kg^−1^ had a stimulating effect on the FA content. An increase in the total FA content was observed due to an increase in the proportion of USFAs. At higher concentrations (100 and 150 mg kg^−1^), the total FA content decreased compared to the control, mainly due to a decrease in the proportion of SFAs, which ensured an increase in the USFA-to-SFA ratio under the influence of zinc. The decrease in the concentrations of C20:0 and C22:0, as well as their absence at a zinc concentration of 150 mg kg^−1^, may suggest a metabolic reorganization toward unsaturated fatty acid production due to changes in the activity of elongase complex enzymes, when zinc toxicity reaches certain levels in lipid metabolism. At the same time, under conditions of excess zinc, an increase in the content of C16:0 was observed alongside a decrease in C14:0. A similar pattern was previously observed by us in wheat leaves under the influence of cadmium [55] and in the hyperaccumulator *Noccaeacaerulescens* [56]. It has been suggested that C14:0 may be used in the elongation process under such conditions. A high C16:0 content in plants exposed to heavy metals has been noted in a number of studies [34,36,37,55], which may be due to the important role of this acid as a precursor for the synthesis of a number of acids [57]. In addition, an increase in the content of C22:1 and C20:1 was observed alongside a decrease in C18:2. A similar trend was noted in a study of *Brassica juncea* seeds after cultivation in excess cadmium [58]. This may be due to the fact that in *B. juncea* seeds, these acids (20:1 and 22:1) are synthesized from oleoyl-CoA by reductases during the elongation process, while oleoyl-CoA is also necessary for the synthesis of C18:2 during desaturation [59]. It is likely that under the influence of heavy metals, elongation processes predominate over desaturation. The general tendency toward the accumulation of USFAs may be associated with their multifunctional role in cells, such as maintaining membrane permeability and indirectly protecting cells from reactive oxygen species, as precursors for the formation of secondary metabolites [60]. USFAs are typically stored as triacylglycerides in the *sn-1* position, which gives them a high energy value and makes them readily available for energy-dependent processes under changing environmental conditions. This allows fast-growing plants to respond to stress-induced conditions [61]. It is worth noting that the changes in FA composition in *S. alba* seeds differed from those in the leaves of the same plant grown under conditions with Zn excess, as shown by Repkina et al., 2023 [30]. In particular, there was a decrease in the ratio of USFAs to SFAs due to an increase in the total SFA content.

The processing of plants grown in contaminated areas remains an important and unresolved issue. In the present study, seeds obtained from *S. alba* plants grown under conditions ofZn excess met all the chemical composition requirements set out by SanPiN except for the Zn concentration, and they contained a higher amount of triglycerides and a high content of essential fatty acids. Overall, based on the biochemical analysis data and the increased zinc content, an important microelement for animals and humans, the seeds can be utilized as an additive in animal feed or human food. However, the accumulation of erucic acid makes them unattractive from a food industry perspective. It has previously been shown that erucic acid consumption caused myocardial lipidosis by affecting the mitochondrial metabolism. Due to the long-chain structure of erucic acid, the oxidation rate in mitochondria is prolonged, as a result of which, TAG accumulates [62]. In addition, erucic acid induces the peroxisomal β-oxidation rate and suppresses the mitochondrial β-oxidation rate, thereby progressing hepatic steatosis and insulin resistance [63]. Nowadays, many countries have standards regulating the maximum erucic acid content in edible oils at 5% to 2%. Additionally, the tolerable daily intake of erucic acid has been established as 7 mg kg^−1^ bw^−1^ [62,64]. If this value is exceeded, using the oil for industrial purposes is considered instead.

Research on *S. alba* has demonstrated the potential of its seeds for biofuel production [40,65,66,67]. *S. alba* seeds have been shown to meet the requirements of the European standard EN 14214, which applies to biofuels, for a number of parameters determining the physicochemical properties of biofuels. Therefore, it was important to evaluate how these characteristics change in plant seeds when grown under conditions of Zn excess. A number of authors [39,40,68] have proposed a mathematical model based on the FA content; the ratios of mono-, di-, and polyunsaturated fatty acids; and the length of the FA carbon chain. The LCV (lowest calorific value) indicator, which provides information on the amount of energy released during complete combustion of the fuel, remains virtually unchanged, at over 38,000 kj kg^−1^, depending on the Zn concentration. Low values are typical for vegetable oils compared to crude oil, resulting in higher specific fuel consumption. However, the experimental values are consistent with data from Sáez-Bastante [40] and exceed those calculated for other vegetable oils (sunflower, olive, palm, etc.). The CN parameter allows one to assess the combustion quality: fuels with a low CN typically cause diesel engine knocking and exhibit increased particulate emissions due to incomplete combustion. In our experiments, as the Zn concentration increased, the CN value decreased, but even at a Zn concentration of 150 mg kg^−1^, CN = 64, it complied with the EN 14214 standard, according to which the minimum CN value is 51. The kinematic viscosity coefficient in the control plants (ν = 6.42 mm^2^ s^−1^) was slightly higher than the upper limit specified in the European standard (ν = 5 mm^2^ s^−1^, EN 14214). At the same time, in the American standard (ASTM D6751), the upper limit for ν is 6 mm^2^ s^−1^. It is worth noting that as the Zn concentration increases, the ν values decrease at a Zn concentration of 150 mg kg^−1^ν = 5.86 mm^2^ s^−1^, which already complies with the American standard. Fuel viscosity also affects injection and combustion. A higher viscosity results in greater resistance in the high-pressure fuel pump, resulting in higher injection pressures and volumes, especially at low engine operating temperatures. The flash point (FP), in °C, determines the safe storage and handling of a substance. Biodiesel has a higher flash point than petrodiesel, making it less hazardous to handle. However, standard EN 14214 sets the lower flash point limit at 122 °C, as a lower flash point may indicate the presence of methanol in biodiesel. Our study demonstrated that despite an increase in the substrate’s Zn concentration, FP values decreased but remained above 200 °C, which meets the standard. CFPP is the temperature at which fuel causes filter plugging due to its crystallization [69]. According to calculations based on the FA composition of seeds, Zn excess does not degrade the expected physicochemical parameters of biofuel; moreover, at a concentration of 150 mg kg^−1^, the kinematic viscosity coefficient decreased. Seeds obtained from plants grown under such conditions can be considered as a feedstock for biofuel.

## 4. Materials and Methods

### 4.1. Plant Material and Growth Conditions

Seeds of yellow mustard (*S. alba*, cv. Belgia) were acquired from the Federal Research Center N.I. Vavilov All-Russian Institute of Plant Genetic Resources (VIR), Ministry of Science and Higher Education, Russia. This study was conducted at the research base of the Agricultural Experiment Station (61°45′6″ N, 119°25′10.9″ E) (WGS84), Petrozavodsk, Republic of Karelia, Russia. On the first day prior to sowing, the seeds were steeped in distilled water. Two day-old seedlings were placed in pots (12 seedlings per a pot), each (height 40 cm, diameter 20 cm) containing 5 kg of pure quartz sand. Appropriate amounts of sulfate salt of Zn (ZnSO_4_ × 7H_2_O) were added to the sand to maintain the required level of Zn (5 (control), 50, 100, and 150 mg kg^−1^) in the substrate and then mixed in order to ensure a uniform distribution of elements. These concentrations are in comparison to the optimum, and are two-, four- and sixfold higher than the maximum allowable concentration (MAC) in sand. MAC standards were published by the Ministry of Agriculture (GN 2.1.7.2041-06 from 2006) [70]. During the growing period, pots were irrigated with Hoagland–Arnon nutrient solution without any added Zn. The pH of the solution was maintained at 6. All pots were placed in a greenhouse under natural day/night conditions with a day/night temperature of 21/14 ± 2 °C and 77 ± 5% relative humidity during the growing period. Plants were harvested at the BBCH 87 stage (70% of pods ripe, seeds dark and hard). The experimental design was completely randomized with three replications for each treatment. The experiment was repeated in the years 2022 and 2023. The pod and seed numbers were assessed for each plant (30 plants per treatment) and then averaged for each treatment.

The germination rate of seeds obtained from experiments with different zinc concentrations was assessed. For this, 25 seeds were placed in Petri dishes (four dishes for each treatment) and distilled water was added. The dishes were placed in a thermostat at a temperature of 22 ± 1 °C. Seeds were examined daily and considered germinated when the root became visible. The number of germinated seeds was calculated 3 (germination rate) and 6 (germination ability) days after sowing and expressed as a percentage, according to Russian national standard No.9670-61.

### 4.2. Trace Elements Assay

A mass of 0.1 g of the seeds (mixed sample from 30 plants) from each concentration was collected. The samples were dried at 105 °C for 1 h and then degraded in a homogenizer. A sample (0.050 g) was placed in a fluoroplastic beaker. Acids (3 mL HNO_3_, 0.4 mL HCl and 0.15 mL HF) were added to each sample, and the mixture was kept at room temperature for 12 h. Then, 1 mL 33% H_2_O_2_ was added to the mixture, after which the samples were heated in an EXPEC 790S (Berghof, Eningen, Germany) microwave digestion system under the following modes: 1—pre-pressure 40 bar, heating time 15 min, target temperature 160 °C, hold time 30 min; 2—pre-pressure 40 bar, heating time 5 min, target temperature 180 °C, hold time 60 min. The samples were then cooled to room temperature and 0.075 g of crystalline H_2_BO_3_ was added to bind HF. They were then heated again in the EXPEC 790S system at a pre-pressure of 40 bar for a heating time of 8 min, and a target temperature of 190 °C, with a hold time of 10 min. The samples were then cooled again at room temperature and transferred to plastic tubes of known weight. The solution weight was adjusted to 40 g with deionized water. Samples were analyzed using inductively coupled-plasma mass spectrometry (ICP-MS) (Agilent 7900, Santa Clara, CA, USA) with the standard settings for this equipment. High-purity standards (standard A and B) (HPS, Charleston, WV, USA) were used for calibration. The data were expressed in mg kg^−1^.

### 4.3. Lipid Extraction and Analysis

The total lipids (TL) from 0.2 g of seeds (mixed sample from 30 plants) were extracted using the Folch method with a chloroform–methanol (2:1 *v*/*v*) mixture [71]. In brief, 20 mL of the samples was filtered using Red Ribbon filter paper (pore size 5–8 μm), after which the precipitate on the filter was washed with 10 mL of pure chloroform. To remove water-soluble impurities, 10 mL of purified deionized water (Simplicity, Millipore, Merck, Darmstadt, Germany) was added to the filtered mixture and left for 2 h in separating funnels (Schott Duran, Mainz, Germany) until the organic phases were completely separated. Lipids remained in the lower chloroform layer, whereas non-lipid substances moved to the upper aqueous methanol phase. Then, the chloroform layer was withdrawn to evaporate under a vacuum on a rotary evaporator (Hei-VAP Advantage HL/G3 (Heidolph, Schwalbach, Germany)) and dried in a vacuum over phosphoric anhydride to a constant weight. The dried samples were re-dissolved with chloroform–methanol (1:1, *v*/*v*) and stored at −20 °C until further processing.

Qualitative and quantitative analyses of individual lipid classes—total phospholipids; mono-, di-, and triacylglycerols; cholesterol; cholesterol esters; and free fatty acids—were performed using high-performance thin-layer chromatography (HPTLC). Total lipids were separated into fractions on ultrapure glass support plates (HPTLC Silicagel 60 F254 Premium Purity (Merck, Germany)). The eluent was a hexane/diethyl ether/acetic acid solvent system (32:8:0.8 *v*/*v*/*v*). The lipid components were determined in the chamber of a TLC Scanner 4 scanning densitometer (CAMAG, Muttenz, Switzerland) under deuterium lamp light at a wavelength of 350 nm. Lipid classes were identified using standard solutions of the following reference standards: a phospholipid mixture (Supelco, Bellefonte, PA, USA), cholesterol (Sigma-Aldrich Co., St. Louis, MO, USA), glyceryl trioleate (Sigma-Aldrich Co., USA), and cholesteryl palmitate (Sigma-Aldrich Co., USA). Samples of total lipids were analyzed in two replicates. The total lipid content was expressed as weight % of tissue dry weight, and the lipid class content was expressed as weight % of total lipid dry weight.

### 4.4. Fatty Acid Analysis

The qualitative and quantitative fatty acid (FA) profile of the TL was analyzed using gas–liquid chromatography (GC) with a mass-selective detector (MS). We had previously subjected the TL mixture to acid methylation. To obtain the fatty acid methyl esters (FAMEs) from the TLs, 0.2 mL of a solution of TLs, 2 mL of methanol, and 0.2 mL of chlorate acetyl (CH_3_COCl) as a catalyst were added to a glass retort (Schott Duran, Mainz, Germany). The obtained solution was heated for 90 min at a fixed temperature of 70–80 °C. After methylation (followed by cooling), 5 mL of hexane was added to each sample. For phase separation, 2 mL of deionized water was poured into each glass retort and transferred into the separatory glass funnels for 15 min. FAMEs remained in the upper hexane layer while residue substances were concentrated in the lower aqueous phase. The hexane layer was evaporated using a rotary evaporator (Hei-VAP Advantage HL/G3 (Heidolph, Schwabach, Germany)) to collect pure FAMEs. Then, 1 mL of hexane for GC (Sigma Aldrich, St. Louis, MO, USA) was added to the collected FAMEs in glass retorts, and the gathered and final solutions were accumulated in GC vials for the following GC analysis.

FAMEs were separated using GC with a mono-quadrupole mass-selective detector (α-Maestro (Saitegra, Moscow, Russia)). The FAs were separated in a gradient thermal configuration (t_start_ = 140 °C—hold for 5 min; increase in t from 140 °C to 240 °C at a rate of 4 °C per min; and t_final_ = 240 °C—hold for 2 min) with an HP-88 (60 m × 0.25 mm × 0.20 µm) capillary column (Agilent Technologies, Santa Clara, CA, USA) using helium as a mobile phase. FAMEs were detected in SCAN and SIM modes. The SCAN mode was used for searching and identifying FAs (qualitative analysis) with scan parameters of 50 to 400 mz^−1^. The SIM mode was used for FAs according to analytical standards with Supelco 37 (Sigma Aldrich, St. Louis, MO, USA), with subsequent quantitative analysis of the components that make up the mixture. The individual FAs in the samples were identified and quantified using the calibration method. The Supelco 37 mixture was used as the reference standard (Sigma Aldrich, St. Louis, MO, USA). The data were analyzed using Maestro Analytic v. 1.026 software (Saitegra, Moscow, Russia) with the NIST library.

### 4.5. Biodiesel Property Predictions

Several biodiesel properties were predicted using mathematical models according to [39,40], which were based on the chemical properties of the hydrocarbon chain. The base parameters for the model were calculated as follows:

1. Average length of chain (LC): LC = Σ(n*Cn)/100, where n is the number of carbon atoms of fatty acid chain of methyl ester and Cn is the weight percentage of each methyl ester containing this FA.
2. Total unsaturation degree (TUD): TUD = (1%Mu + 2%Du + 3%Tu)/100%, where %Mu is the percentage in weight of monounsaturated methyl esters, %Du is the percentage in weight of diunsaturated methyl esters, and %Tu is the percentage in weight of triunsaturated methyl esters.
3. Low calorific value (LCV, kj kg^−1^): LCV = 29385.4 + 486.866 * LC − 387.766 * TUD.
4. Cetane number (CN): CN = 46.6632 − 1.7357 * LC +12.3976 * TUD + 0.243275 * LC^2^ − 2.64964 * LC * TUD + 5.65655 * TUD^2^.
5. Kinematic viscosity at 40 °C (µ, mm^2^ s^−1^): µ = −1.8327 + 0.209794 * LC + 0.738911 * TUD + 0.0166791 * LC^2^ − 0.16336 * LC * TUD + 0.335547 * TUD^2^.
6. Flash point (FP, °C): FP = 1008.48 − 136.166 * LC + 142.578 * TUD + 5.14811 * LC^2^ − 10.6906 * LC * TUD + 9.26352 * TUD^2^.
7. Cold filter plugging point (CFPP, °C): CFPP = 82.3069 − 17.831 * LC + 50.9813 * TUD + 0.8584 * LC^2^ − 5.5148 * LC * TUD + 11.949 * TUD^2^.

### 4.6. Statistical Analysis

Data were subjected to an analysis of variance (one-way ANOVA). The data were processed using Excel 2013 (Microsoft Corp., Redmond, WA, USA) and analyzed with Statgraphics Plus 5.0 (Statgraphics Technologies, Inc., The Plains, VA, USA) statistical software. Data are presented as means ± standard errors. The least significant difference (LSD) test was used to compare means. Differences at *p* ≤ 0.05 were considered statistically significant.

## 5. Conclusions

In this study, an integrated analysis of the seed yield, FA and lipid profile, and modeled biodiesel parameters under Zn stress in *S. alba* was performed. Based on the data obtained, it can be concluded that *S. alba* is capable of growing on Zn-contaminated substrates and producing seeds. The Zn concentration (50 mg kg^−1^) had a slight effect on seed productivity but it stimulated the accumulation of FAs by increasing the proportion of unsaturated FAs. However, high Zn concentrations (100 and 150 mg kg^−1^) negatively affected the seed yield parameters, making them unsuitable for use as seed material. Additionally, excess Zn increased the amount of TAGs in seeds and decreased the total FA content by significantly reducing the proportion of saturated fatty acids. In all of the experimental treatments, the *S. alba* seeds showed elevated erucic acid content, which makes them suitable for processing into oil for technical purposes only (biofuel). Moreover, according to calculations of the parameters determining the estimated physicochemical properties of biofuel, seeds obtained after cultivation on a Zn-contaminated substrate not only did not differ from the control, but also had a better kinematic viscosity coefficient. Therefore, when mustard is grown on a Zn-contaminated substrate, the seeds of these plants can be further processed, and for example, used as a raw material for biofuel production.

## Figures and Tables

**Table 1:** Pod and seed number and thousand-seed weight of *S. alba* grown in the presence of different substrate Zn concentrations.

Parameter	Zn Concentration, mg kg^−1^ Substrate			
	5	50	100	150
Pods number (plant ^−1^)	367 ± 17 a	311 ± 16 a	234 ± 25 b	194 ± 4 b
Seeds number (plant ^−1^)	624 ± 2 a	610 ± 76 a	544 ± 106 a	395 ± 165 a
Thousand-seed weight (g)	8.5 ± 0.3 a	8.0 ± 0.6 a	7.7 ± 0.3 a	5.7 ± 0.3 b

Values are means ± SE (n = 3). Different letters indicate significant differences between treatments (*p* < 0.05), determined by Fisher’s least significant difference (LSD) test.

**Table 2:** Germinating rate and viability (%) of *S. alba* seeds formed under Zn excess in the substrate.

Time (Days)	Zn Concentration, mg kg^−1^ Substrate			
	5	50	100	150
3	96	85	81	72
6	98	93	88	75

**Table 3:** Content of some physiologically important trace elements in *S. alba* seeds formed in the presence of different Zn concentrations in the substrate.

Zn Concentration, mg kg^−1^ Substrate	Content of Trace Elements in Seeds, mg kg^−1^			
	P	Mn	Cu	Zn
5	7621.9 ± 615.4 c	30.1 ± 4.6 b	5.9 ± 1.1 b	47.5 ± 1.1 c
50	9740.1 ± 784.8 b	50.8 ± 7.3 a	6.2 ± 1.1 b	185.3 ± 1.1 b
100	10,738.9 ± 864.7 ab	50.8 ± 7.3 a	5.6 ± 1.1 b	185.9 ± 1.1 b
150	11,065.5 ± 890.9 a	53.5 ± 7.7 a	7.8 ± 1.3 a	283.9 ± 1.1 a
MPV *	-	-	15	50

Values are means ± SE (n = 3). Different letters indicate significant differences between treatments (*p* < 0.05), determined by Fisher’s least significant difference (LSD) test. * Maximum permissible values (MPV) according to the state standard for seeds when used in food safety [38].

**Table 4:** The total lipid content (% per dry weight) in *S. alba* seeds formed under Zn excess in the substrate.

Zn Concentration, mg kg^−1^ Substrate	Lipid Classes										
	PL	MAG	DAG	CH	MGDG	DGDG	FFA	TAG	ECH	Waxes	TAG/PL
5	0.22	0.04	0.05	0.17	0.14	0.03	0.08	1.30	0.31	0.25	5.91
50	0.17	0.04	0.04	0.13	0.14	0.06	0.07	1.30	0.32	0.30	7.65
100	0.17	0.04	0.04	0.14	0.15	0.06	0.06	1.35	0.18	0.28	7.94
150	0.20	0.04	0.06	0.15	0.16	0.10	0.08	1.36	0.34	0.26	6.80
*p*-values	≤0.05	0.05	≤0.05	≤0.05	0.05	≤0.05	≤0.05	≤0.05	≤0.05	≤0.05	

Values (n = 2) are presented as arithmetic means. The standard error was low and was approximately 0.003–0.009. PL—total phospholipids, MAG—monoacylglycerol, DAG—diacylglycerol, CH—cholesterol, MGDG—monogalactosyldiglyceride, DGDG—digalactosyldiglyceride, FFA—free fatty acid, TAG—triacylglycerol, ECH—esterified cholesterol.

**Table 5:** The fatty acid (FA) (µg mL^−1^) content in *S. alba* seeds, formed under Zn excess in the substrate.

Fatty Acids	Zn Concentration, mg kg^−1^ Substrate				*p*-Values
	5	50	100	150	
	Saturated fatty acids (SFAs)				
Lauric acid C12:0	0.36	0.50	0.29	0.46	≤0.05
Myristic acid C14:0	5.47	3.57	3.09	3.50	0.05
Pentadecanoic acid C15:0	2.52	2.40	3.48	2.23	0.05
Palmitic acid C16:0	110.23	129.73	128.91	132.93	≤0.05
Stearic acid C18:0	35.11	40.74	39.34	41.15	≤0.05
Arachidic acid C20:0	932.04	880.17	110.05	-	≤0.05
Behenic acid C22:0	30.10	28.18	20.78	-	≤0.05
Lignoceric acid C24:0	42.13	42.51	39.68	35.92	0.05
Total content	1157.96	1127.80	345.62	216.19	
	Unsaturated fatty acids (USFAs)				
Palmitoleic acid C16:1n-7	6.98	6.92	6.38	7.28	≤0.05
Oleic acid C18:1n-9	673.57	978.85	989.71	991.33	≤0.05
Linoleic acid C18:2n-6	798.99	794.43	774.81	539.58	0.05
Linolenic acid C18:3n-3	374.87	455.03	484.48	417.96	≤0.05
Gadoleic acid C20:1n-9	280.56	417.19	433.99	440.93	≤0.05
Eicosadienoic acid C20:2n-6	34.97	27.48	23.44	24.27	0.05
Erucic acid C22:1n-9	470.55	555.31	567.86	549.04	≤0.05
Nervonic acid C24:1n-9	67.00	69.94	70.40	60.21	≤0.05
9,12-hexadecadienoic acid C16:2n-4	+	+	+	+	
Vaccenic acid C18:1n-7	+	+	+	+	
Paullinic acid C20:1n-7	+	+	+	+	
Total content	2707.49	3305.15	3351.07	3030.60	
USFAs/SFAs	2.34	2.93	9.69	14.03	

“+”—the identified component was not quantified due to its absence in the Supelco37 mixture. Values are presented as arithmetic means; the standard error was low, at approximately 0.003–0.009, as the analysis was performed using GC-MSD (n = 3) with high precision and accuracy.

**Table 6:** Expected physical and chemical properties of biofuels.

Zn Concentration, mg kg^−1^ Substrate	LC	UD	LCV, kj kg^−1^	CN	ν, mm^2^ s^−1^	FP, °C	CFPP, °C
5	19.7	1.12	38,542.36	70.98	6.42	259.31	−14.58
50	19.2	1.14	38,291.17	66.51	6.05	232.47	−9.33
100	19	1.17	38,182.17	64.85	5.87	221.64	−6.81

LC—fatty acid chain length; UD—unsaturation degree; LCV—low calorific value; CN—cetane number; ν—kinematic viscosity; FP—flash point; CFPP—cold filter plugging point.

## Data Availability

All data and tables in this manuscript are original.
